# Persistently high venous-to-arterial carbon dioxide differences during early resuscitation are associated with poor outcomes in septic shock

**DOI:** 10.1186/cc13160

**Published:** 2013-12-13

**Authors:** Gustavo A Ospina-Tascón, Diego F Bautista-Rincón, Mauricio Umaña, José D Tafur, Alejandro Gutiérrez, Alberto F García, William Bermúdez, Marcela Granados, César Arango-Dávila, Glenn Hernández

**Affiliations:** 1 Intensive Care Unit, Fundación Valle del Lili, Av. Simón Bolívar Cra. 98, Cali, Colombia; 2 Biomedica Research Group, Universidad ICESI, Cali, Colombia; 3 Departamento de Medicina Intensiva, Pontificia Universidad Católica de Chile, Santiago, Chile

## Abstract

**Introduction:**

Venous-to-arterial carbon dioxide difference (Pv-aCO_2_) may reflect the adequacy of blood flow during shock states. We sought to test whether the development of Pv-aCO_2_ during the very early phases of resuscitation is related to multi-organ dysfunction and outcomes in a population of septic shock patients resuscitated targeting the usual oxygen-derived and hemodynamic parameters.

**Methods:**

We conducted a prospective observational study in a 60-bed mixed ICU in a University affiliated Hospital. 85 patients with a new septic shock episode were included. A Pv-aCO2 value ≥ 6 mmHg was considered to be high. Patients were classified in four predefined groups according to the Pv-aCO2 evolution during the first 6 hours of resuscitation: (1) persistently high Pv-aCO2 (high at T0 and T6); (2) increasing Pv-aCO2 (normal at T0, high at T6); (3) decreasing Pv-aCO2 (high at T0, normal at T6); and (4) persistently normal Pv-aCO2 (normal at T0 and T6). Multiorgan dysfunction at day-3 was compared for predefined groups and a Kaplan Meier curve was constructed to show the survival probabilities at day-28 using a log-rank test to evaluate differences between groups. A Spearman-Rho was used to test the agreement between cardiac output and Pv-aCO2. Finally, we calculated the mortality risk ratios at day-28 among patients attaining normal oxygen parameters but with a concomitantly increased Pv-aCO2.

**Results:**

Patients with persistently high and increasing Pv-aCO_2_ at T6 had significant higher SOFA scores at day-3 (p < 0.001) and higher mortality rates at day-28 (log rank test: 19.21, p < 0.001) compared with patients who evolved with normal Pv-aCO_2_ at T6. Interestingly, a poor agreement between cardiac output and Pv-aCO_2_ was observed (r^2^ = 0.025, p < 0.01) at different points of resuscitation. Patients who reached a central venous saturation (ScvO)_2_ ≥ 70% or mixed venous oxygen saturation (SvO_2_) ≥ 65% but with concomitantly high Pv-aCO_2_ at different developmental points (i.e., T0, T6 and T12) had a significant mortality risk ratio at day-28.

**Conclusion:**

The persistence of high Pv-aCO_2_ during the early resuscitation of septic shock was associated with more severe multi-organ dysfunction and worse outcomes at day-28. Although mechanisms conducting to increase Pv-aCO2 during septic shock are insufficiently understood, Pv-aCO_2_ could identify a high risk of death in apparently resuscitated patients.

## Introduction

Inadequate tissue perfusion is a pivotal factor in the pathogenesis and clinical course of multiorgan failure in the critically ill [1]. Current techniques for monitoring tissue perfusion have largely focused on systemic blood flow and the balance between oxygen demand and supply [2,3]. An early hemodynamic optimization that targets central venous oxygen saturation (ScvO_2_) and systemic hemodynamic parameters improves outcomes in severe sepsis and septic shock [4], reinforcing the idea that tissue perfusion abnormalities are flow dependent at least during the very early stages. However, normalizing systemic hemodynamic parameters does not guarantee adequate tissue perfusion [5-7], and in fact a substantial number of patients still progress to multiorgan dysfunction and death despite meeting ScvO_2_ targets [4].

In the past, authors described the coexistence of venous acidemia and increased venous carbon dioxide (CO_2_) during cardiac arrest in both animals [8] and critically ill humans [9]. Thereafter, increases in the venous-to-arterial carbon dioxide difference (Pv-aCO_2_) were reported during hypovolemic, cardiogenic, obstructive, and septic shock [10-12]. Interestingly, an inverse curvilinear relationship between Pv-aCO_2_ and cardiac output was described, highlighting the importance of blood flow on venous CO_2_ accumulation [13,14]. Pv-aCO_2_ thus aroused clinical interest as a marker of global perfusion during shock states, although some studies questioned its prognostic value [14]. In fact, some *in vivo* models evaluating the mechanisms conducting to venous CO_2_ accumulation during non-inflammatory conditions [15-17] challenged the ability of Pv-aCO_2_ to identify tissue dysoxia because it only rises during ischemic hypoxia, but not during hypoxic or anemic hypoxia for comparable declines in oxygen delivery and oxygen consumption. However, more recent data suggest that high Pv-aCO_2_ could identify septic patients who remain inadequately resuscitated despite achieving oxygen metabolism targets, reinforcing the notion of Pv-aCO_2_ as a marker of global perfusion due to its ability to track blood flow alterations [18] or even detect anaerobic CO_2_ generation [19]. Furthermore, patterns of recovery or derangement of Pv-aCO_2_ during very early stages of resuscitation of septic shock have not been widely described and recent studies trying to demonstrate the reliability of Pv-aCO_2_ as a tool in resuscitation of septic patients could have been influenced by selection bias because not all potential patients were elected to catheter insertion and goal-directed therapy [20,21].

Recent publications in critical care demonstrate that oxygen-derived parameters such as ScvO_2_ or mixed venous oxygen saturation (SvO_2_) are commonly normalized at ICU admission [22] and maneuvers such as emergent intubation can quickly improve ScvO_2_ despite regional and tissue perfusion derangements [23]. Because global and regional hypoperfusion have been incriminated in the development of multiple organ failure, investigation on surrogate markers of such phenomenon remains important in critical care. Given that Pv-aCO_2_ can track the adequacy of systemic perfusion during shock states, we sought to test whether the time course of Pv-aCO_2_ during the early phases of resuscitation is related to the development of more severe multiorgan dysfunction and worse outcomes in a population of septic shock patients resuscitated by targeting the usual oxygen-derived and hemodynamic parameters.

## Materials and methods

This prospective observational study was performed in a 60-bed mixed ICU in a university-affiliated hospital. We examined all patients with a new septic shock episode admitted to the emergency room or proceeding from clinical wards during a 24-month period. Septic shock was defined using the criteria of the American College of Chest Physicians and the Society of Critical Care Medicine Consensus Conference [24]. Patients were excluded if they were younger than 18 years old, pregnant, had severe chronic obstructive pulmonary disease (GOLD 3 and 4 categories according to the current classification at the time of our study) or advanced liver cirrhosis (Child–Pugh C).

### General management

All patients admitted to the emergency room or proceeding from clinical wards who fulfilled the diagnosis criteria for septic shock were evaluated by the ICU rapid response team according to our local procedures. Each patient was equipped with an arterial cannula and a pulmonary artery catheter (CCO Swan–Ganz catheter; Edwards Life sciences, Irvine, CA, USA). Our early goal-directed therapy included a bundle of interventions that sought to obtain: mean arterial pressure ≥65 mmHg; urine output ≥0.5 ml/kg/minute; normalization of serum lactate; and ScvO_2_ ≥70% or SvO_2_ ≥65%. The use of vasopressors (dopamine or norepinephrine) was standardized to maintain a mean arterial pressure ≥65 mmHg, and repeated fluid challenges with crystalloids or colloids were used to optimize the stroke volume as well as to allow the lowest dose of vasopressors and pulse-pressure variability <12%. Dobutamine was added for persistent ScvO_2_ ≤70% or SvO_2_ ≤65% after fluid resuscitation. A low dose of hydrocortisone was given within 6 hours of resuscitation when use of vasopressors persisted after an adequate fluid restitution. Mechanical ventilation was provided when needed under light sedation (midazolam) and analgesia (fentanyl); the tidal volume was limited to 6 to 8 ml/kg. Glycemic control was adjusted to maintain glucose levels <150 mg/dl. Finally, stress ulcer and venous thrombosis prophylaxis were provided according to international recommendations [25].

### Study protocol

The Fundación Valle del Lili’s Ethical and Biomedical research committee approved the current study. A written informed consent was waived because no new therapeutic interventions were performed and all measurements and procedures routinely followed the local protocols for the management of severe sepsis and septic shock.

Time 0 (T0) was declared when the pulmonary artery catheter was inserted using common monitoring tracings to place the distal port in the pulmonary artery and the proximal port in the right atrium, approximately 3 cm above the tricuspid valve. In order to standardize T0, we recorded the total volume of fluids administered and the time elapsed between the start of resuscitation (first hypotension episode) and the pulmonary artery catheter insertion (T0).

We collected arterial venous blood samples and central and mixed venous blood samples for arterial–venous gases (ABL 300; Radiometer Copenhagen, Denmark) and arterial lactate measurements at T0, and 6 hours (T6), 12 hours (T12) and 24 hours (T24) later. We simultaneously registered hemodynamic and respiratory variables at each measurement. We defined Pv-aCO_2_ as the difference between the mixed venous CO_2_ partial pressure and the arterial CO_2_ partial pressure. Previous studies considered Pv-aCO_2_ ≥6 mmHg abnormal [14]. Hence, we classified the patients according to the Pva-CO_2_ development during the first 6 hours of resuscitation: persistently high Pv-aCO_2_ (high at T0 and T6); increasing Pv-aCO_2_ (normal at T0, high at T6); decreasing Pv-aCO_2_ (high at T0, normal at T6); and persistently normal Pv-aCO2 (normal at T0 and T6). The Sequential Organ Failure Assessment score [26] was used to describe multiorgan dysfunction at day 3 and we also described mortality at day 28 for the pre-defined groups.

### Data analysis

After exclusion of a normal distribution of the data by the Kolmogorov–Smirnov test, we used a Kruskal–Wallis test to compare continuous variables (followed by Bonferroni correction for multiple comparisons) and *a chi-squared test* (or Fisher’s exact test, when appropriate) for discrete variables. Survival probabilities at day 28 were described using a Kaplan–Meier curve and differences between groups were calculated using a log-rank test before and after adjusting for SvO_2_ at T6. The development of SvO_2_, ScvO_2_, lactate, cardiac output, mean arterial pressure and Pv-aCO_2_ during the first 24 hours were analyzed using a repeated-measures analysis of variance. Spearman’s rho was used to test the agreement between cardiac output and Pv-aCO_2_. We also calculated the mortality risk ratios at day 28 in patients who attained ScvO_2_ ≥70% or SvO_2_ ≥65% but maintained persistently high Pv-aCO_2_ at different points during resuscitation (T0, T6, and T12). Data were expressed as medians and 25 to 75% interquartile ranges. *P* ≤0.05 (two-tailed) was considered significant.

## Results

During the 24-month period, 108 patients older than 18 years with a new episode of septic shock were screened. Patients with advanced cirrhosis (*n* = 4), patients with severe chronic obstructive pulmonary disease (*n* = 8) and pregnant women (*n* = 4) were not included for analysis; additionally, a central catheter could not be placed in four patients, and three refused the procedure. The final sample was therefore 85 patients. The median length of ICU stay for all patients was 6 days (25 to 75% interquartile range, 3 to 11 days), and the 28-day mortality rate was 37.6%. The median time elapsed from sepsis-induced hypotension to catheter insertion was 3.0 hours (25 to 75% interquartile range, 1.0 to 4.0 hours) and the median volume of fluids received before catheter insertion was 2,079 ml (25 to 75% interquartile range, 1,184 to 3,135 ml) for all patients.

Thirty-six patients had Pv-aCO_2_ <6.0 mmHg at T0 and T6, and 17 patients had a high PvaCO_2_ at T0 but it fell below 6 mmHg at T6 (a total of 53 patients had Pv-aCO_2_ <6 mmHg at T6); on the other hand, 24 patients had a persistently high Pv-aCO_2_ during the first 6 hours and the remaining eight evolved from normal at T0 to high PvaCO_2_ at T6 (32 patients had Pv-aCO_2_ ≥6 mmHg at T6).

**Figure 1:**
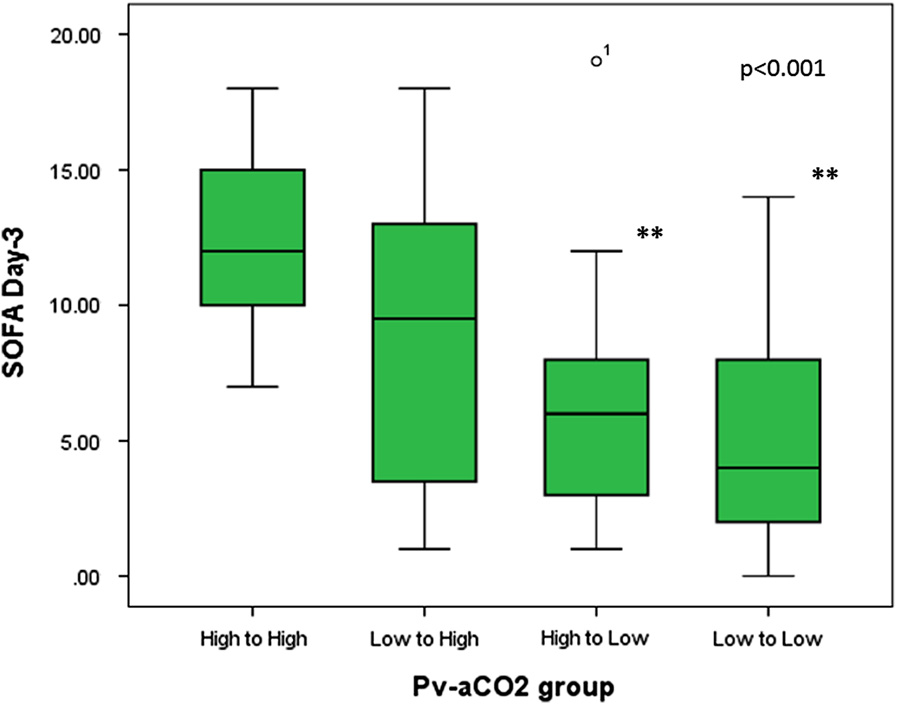
**Sequential Organ Failure Assessment scores at day 3 by the early development (first 6 hours) of mixed venous-to-arterial carbon dioxide difference.** Kruskal–Wallis test, *P* <0.001. **Significant differences between persistently high mixed venous-to-arterial carbon dioxide difference (Pv-aCO_2_) and low Pv-aCO_2_ group; and between persistently high Pv-aCO_2_ and decreasing Pv-aCO_2_ group after Bonferroni correction. H-H, Pv-aCO_2_ high at Time 0 (T0) and 6 hours later (T6); L-H, Pv-aCO_2_ normal at T0 and high at T6; H-L, Pv-aCO_2_ high at T0 and normal at T6; and L-L, Pv-aCO_2_ normal at T0 and T6. SOFA, Sequential Organ Failure Assessment.

**Figure 2:**
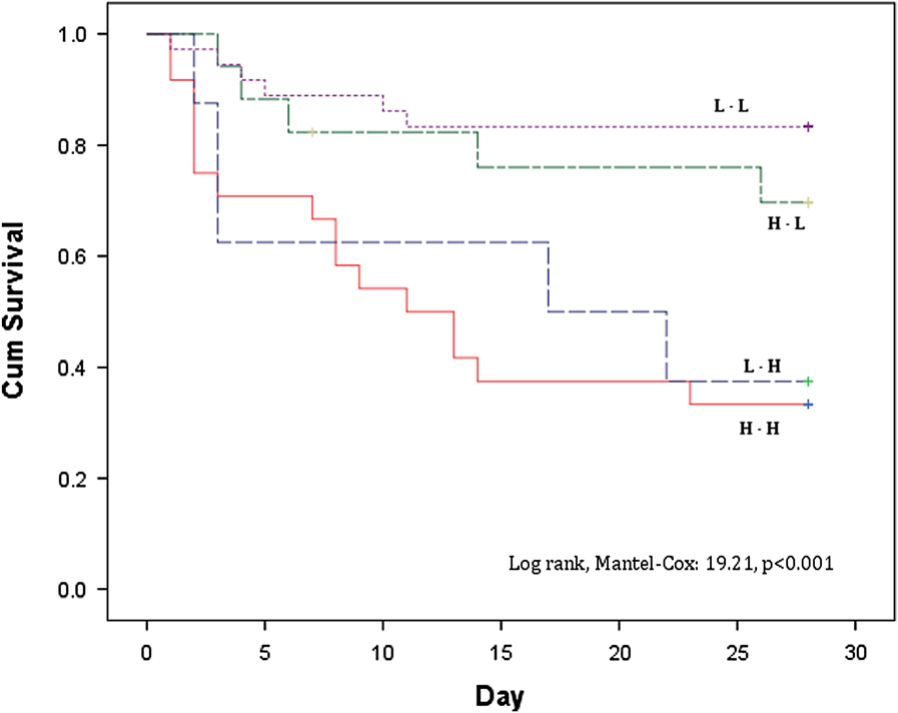
**Survival probabilities at day 28 by the development of mixed venous-to-arterial carbon dioxide difference during the first 6 hours of resuscitation.** Log-rank, Mantel–Cox: 19.21, *P* <0.001. H-H, mixed venous-to-arterial carbon dioxide difference (Pv-aCO_2_) high at Time 0 (T0) and 6 hours later (T6); L-H, Pv-aCO_2_ normal at T0 and high at T6; H-L, Pv-aCO_2_ high at T0 and normal at T6; and L-L, Pv-aCO_2_ normal at T0 and T6.

**Figure 3:**
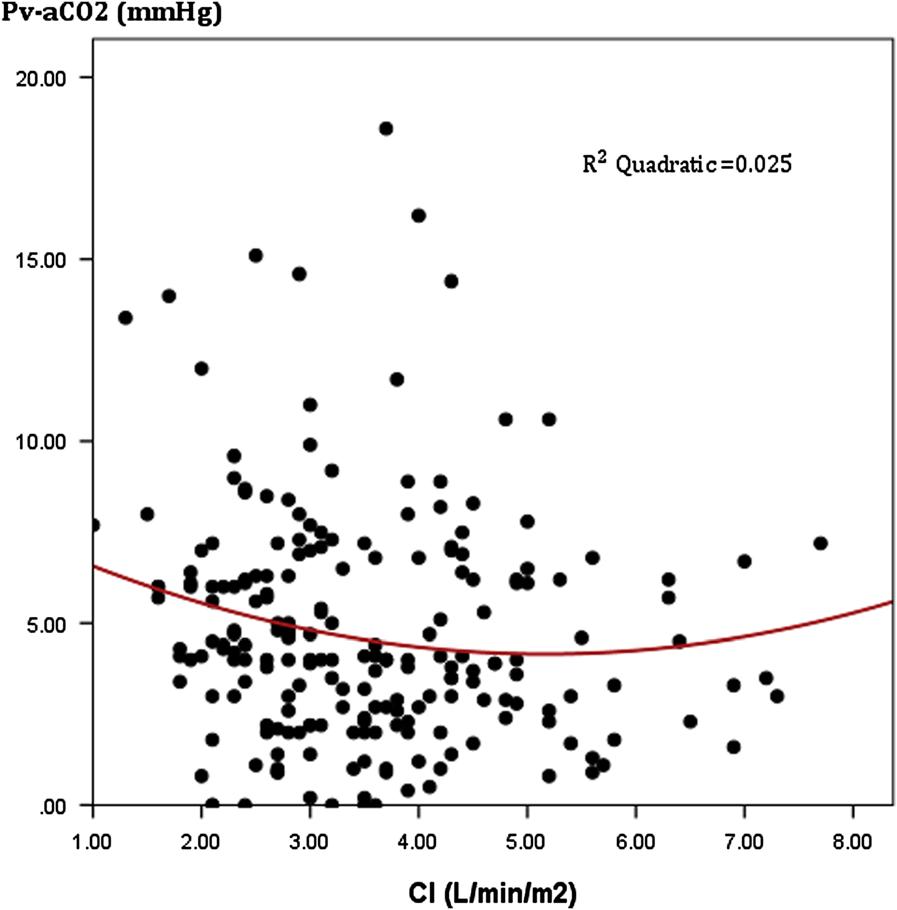
**Scatter plot between cardiac index and mixed venous-to-arterial carbon dioxide difference.** All patients at Time 0 (T0) and 6 hours (T6), 12 hours (T12) and 24 hours (T24) later. Pearson correlation: 0.16; *r*^2^ = 0.025; *P* <0.01. CI, confidence interval; Pv-aCO_2_, mixed venous-to-arterial carbon dioxide difference.

We did not find any significant difference with regard to Acute Physiology and Chronic Health Evaluation II score, comorbidities, demographics, or respiratory and hemodynamic variables between groups (Tables 1 and 2), and neither for the volume of fluids received before inclusion (T0). Doses of vasopressors or inotropic received were similar for the groups both at T0 and T6 (Table 2). Multiorgan dysfunction at day 3 was significantly higher among patients with persistently high Pv-aCO_2_ compared with those with persistently normal or decreasing Pv-aCO_2_ during the first 6 hours of resuscitation (Kruskal–Wallis test, *P* <0.001) (Figure 1). Likewise, patients with persistently high Pv-aCO_2_ during the first 6 hours of resuscitation had a significant lower survival at day 28 compared with those who normalized Pv-aCO_2_ during this period (log-rank, Mantel–Cox: 19.21, *P* <0.001; Figure 2). These results were maintained after adjusting for the SvO_2_ achieved at T6 (log-rank test, *P* <0.001). The time course of SvO_2_, ScvO_2_ and cardiac output did not significantly differ between Pv-aCO_2_ groups (Table 2) nor between survivors and nonsurvivors at day 28 (Figures S1a,b and S2 in Additional file 1). Interestingly, a poor agreement between cardiac output and Pv-aCO_2_ was observed both at each time of resuscitation and when all data were pooled (*r*^2^ = 0.025, *P* <0.01) (Figure 3; Figure S3 in Additional file 1).

**Table 1:** Patient characteristics

**Variable**	**Group 1, H-H**	**Group 2, L-H**	**Group 3, H-L**	**Group 4, L-L**	***P *** **value**
	**(*n*** **= 24)**	**(*n*** **= 8)**	**(*n*** **= 17)**	**(*n*** **= 36)**	
Age (years)	63.0 (54.7 to 75.0)	63.5 (50.5 to 76.5)	55.0 (53.0 to 76.0)	62.0 (49.3 to 71.8)	0.83
Gender, male (%)	17 (70.8)	4 (50.0)	10 (58.8)	22 (61.1)	0.72
APACHE II	24.4 (21.2 to 26.0)	25.2 (21.0 to 27.0)	23.4 (21.2 to 25.6)	24.8 (22.2 to 25.8)	0.11
Time between diagnosis and catheter insertion (T0)	2.0 (2.0 to 4.0)	1.0 (1.0 to 3.3)	3.0 (1.0 to 4.0)	3.0 (2.0 to 4.0)	0.44
Fluids received before catheter insertion	2,039 (1,343 to 2,834)	1,934 (849 to 4,575)	2,500 (1,430 to 3,628)	2,000 (1,025 to 3,153)	0.76
Temperature (°C)	37.5 (37.2 to 37.9)	37.4 (37.1 to 37.9)	37.6 (37.0 to 38.0)	37.4 (37.4 to 37.8)	0.74
Hemoglobin (g/dl)	9.4 (8.7 to 11.6)	8.8 (7.4 to 11.4)	9.8 (9.1 to 11.4)	9.9 (9.1 to 11.4)	0.47
Source of infection, *n* (%)					
Pneumonia	9	3	4	10	
Abdominal	8	4	6	14	
Urinary	2	1	3	4	
Soft tissue	2	0	1	2	
No specific site	3	0	2	4	
Other	0	0	1	2	
Culture positive, *n* (%)	20 (83.3)	5 (62.5)	12 (70.6)	19 (52.8)	0.11
Antibiotics given at T0, *n* (%)	24 (100)	7 (87.5)	16 (94.1)	32(88.9)	0.38
Antibiotics adequate, *n* (%)	23 (95.8)	7 (87.5)	16 (94.1)	27 (75.0)	0.10
Hydrocortisone, *n* (%)	22 (91.7)	8 (100)	15 (88.2)	31 (86.1)	0.68
Transfusion RBC, *n* (%)	4 (16.7)	3 (37.5)	3 (17.6)	4 (11.1)	0.35
Fluids and vasoactive agents					
Fluids (ml), IQ 25 to 75					
T0	2,039 (1,343 to 2,834)	1,934 (849 to 4,575)	2,500 (1,430 to 3,628)	2,000 (1,025 to 3,153)	0.76
T6	4,733 (3,196 to 6,360)	4,082 (1,576 to 7,584)	4,845 (2,550 to 6,560)	4,673 (3,380 to 7,348)	0.83
Norepinephrine (μg/kg/minute), IQ 25 to 75, *n*					
T0	0.25 (0.19 to 0.36), 24	0.29 (0.14 to 1.20), 6	0.19 (0.10 to 0,37), 16	0.23 (0.10 to 0.38), 30	0.81
T6	0.20 (0.11 to 0.40), 24	0.36 (0.09 to 0.49), 6	0.12 (0.11 to 0.18), 15	0.19 (0.08 to 0.39), 31	0.45
Dopamine (μg/kg/minute), IQ 25 to 75, *n*					
T0	–	7.1 (7.1 to 7.3), 2	10.7 (10.7 to 10.7), 1	5.1 (2.8 to 6.6), 5	0.14
T6	–	–	8.0 (8.0 to 8.0), 1	5.3 (2.5 to 6.4), 4	0.16
Dobutamine (μg/kg/minute), IQ 25 to 75, *n*					
T0	3.1 (2.7 to 4.5), 5	–	5.9 (5.1 to 6.7), 2	3.3 (3.0 to 4.2), 3	0.15
T6	3.1 (2.7 to 5.6), 3	–	5.1 (4.7 to 6.3), 4	3.8 (3.3 to 4.8), 3	0.21

*H-H*, venous-to-arterial carbon dioxide difference (Pv-aCO_2_) high at T0 and T6; *L-H*, Pv-aCO_2_ normal at T0 and high at T6; *H-L*, Pv-aCO_2_ high at T0 and normal at T6; and L-L, Pv-aCO_2_ normal at T0 and T6. APACHE, Acute Physiology and Chronic Health Evaluation; IQ 25 to 75, 25 to 75% interquartile range; RBC, red blood cells; T0, time 0; T6, 6 hours after Time 0.

**Table 2:** Hemodynamic, oxygen and ventilatory parameters

**Variable**	**Group 1, H-H**	**Group 2, L-H**	**Group 3, H-L**	**Group 4, L-L**	***P *** **value**
	**(*n*** **= 24)**	**(*n*** **= 8)**	**(*n*** **= 17)**	**(*n*** **= 36)**	
**Hemodynamic variables**					
Heart rate (beats/minute)					
T0	112 (103 to 129)	111 (77 to 138)	104 (86 to 126)	109 (93 to 118)	0.31
T6	114 (101 to 125)	103 (73 to 127)	98 (85 to 114)	103 (88 to 118)	0.14
Cardiac index (l/minute/m^2^)					
T0	3.0 (2.3 to 4.2)	3.6 (2.7 to 4.6)	3.0 (2.5 to 5.0)	3.5 (2.7 to 4.5)	0.63
T6	3.2 (2.2 to 4.3)	3.3 (2.4 to 3.8)	3.7 (2.2 to 5.5)	3.6 (2.8 to 4.3)	0.75
MAP (mmHg)					
T0	65.0 (61.0 to 72.8)**	67.0 (65.3 to 69.5)	73.0 (68.0 to 82.5)**	72.0 (64.5 to 77.0)	0.02
T6	69.0 (65.3 to 75.8)	77.5 (70.5 to 96.0)	68.0 (63.5 to 72.0)	71.5 (65.0 to 78.8)	0.06
CVP (mmHg)					
T0	14.5 (10.3 to 17.0)	10.0 (9.0 to 12.8)	12.0 (8.5 to 15.0)	10.0 (7.0 to 14.0)	0.10
T6	13.5 (10.0 to 15.8)**	15.0 (10.0 to 17.0)	10.0 (5.0 to 12.5)**	10.0 (7.3 to 13.0)	0.03
PAOP (mmHg)					
T0	17.0 (14.0 to 20.0)	12.5 (10.0 to 15.0)	17.0 (12.0 to 22.0)	15.0 (11.0 to 20.0)	0.52
T6	19.0 (15.0 to 24.0)**	14.5 (10.0 to 19.0)	13.0 (10.0 to 15.0)**	16.0 (12.0 to 20.0)	0.03
**Blood gases and oxygen variables**					
pH					
T0	7.30 (7.18 to 7.37)	7.31 (7.22 to 7.40)	7.34 (7.22 to 7.41)	7.32 (7.24 to 7.40)	0.85
T6	7.32 (7.20 to 7.36)	7.35 (7.30 to 7.42)	7.35 (7.26 to 7.39)	7.34 (7.30 to 7.39)	0.33
PaCO_2_ (mmHg)					
T0	30.5 (24.1 to 36.6)	34.2 (24.7 to 36.9)	31.0 (23.6 to 33.6)	27.5 (23.7 to 36.5)	0.81
T6	26.1 (22.4 to 32.7)	31.0 (21.6 to 34.0)	31.3 (26.8 to 33.6)	27.5 (21.7 to 32.1)	0.25
PvmCO_2_ (mmHg)					
T0	40.6 (33.9 to 46.5)^**¶**^	29.5 (25.0 to 35.3)	39.7 (35.5 to 47.1) 34.3	32.8 (27.3 to 40.9)^**¶**^	0.03
T6	38.0 (29.9 to 39.7)^**¶**^	38.3 (28.0 to 46.2)	(30.4 to 36.8)	30.0 (25.5 to 35.5)^**¶**^	0.02
PvcCO_2_ (mmHg)					
T0	38.2 (29.1 to 50.5)	40.1 (34.2 to 43.4)	36.7 (35.5 to 47.1) 33.9	32.5 (28.1 to 38.4)	0.12
T6	33.7 (28.3 to 36.9)	33.8 (31.4 to 41.4)	(30.4 to 36.8)	29.0 (25.1 to 35.8)	0.20
PaO_2_ (mmHg)					
T0	112.2 (79.3 to 167.3)	117.1 (77.5 to 139.5)	115.7 (81.4 to 140.8)	100 (86.0 to 123.9)	0.70
T6	113.6 (83.0 to 147.3)	132.5 (109.8 to 197.8)	105.0 (83.5 to 127.4)	111.5 (86.2 to 135.7)	0.34
PaO_2_/FiO_2_ (mmHg)					
T0	188.9 (117.1 to 265.0)	166.9 (143.8 to 244.1)	233.7 (184.6 to 314.0)	214.3 (141.6 to 368.0)	0.28
T6	177.9 (136.4 to 298.4)	265.0 (185.5 to 383.5)	239.3 (185.1 to 306.3)	253.5 (181.6 to 368.0)	0.17
Lactate (mmol/l)					
T0	4.3 (2.0 to 7.9)	2.9 (2.0 to 9.7)	2.7 (1.5 to 3.9)	2.9 (1.7 to 4.7)	0.16
T6	3.3 (2.1 to 6.8)**^¶^	2.9 (1.1 to 7.1)	1.3 (0.9 to 2.3)**	2.0 (1.0 to 3.5)^**¶**^	0.002
ScvO_2_ (%)					
T0	64.0 (54.7 to 75.1)	66.8 (59.7 to 74.5)	73.0 (70.8 to 76.7)	67.1 (62.1 to 75.9)	0.36
T6	70.8 (66.4 to 73.5)	73.4 (69.3 to 78.0)	72.3 (67.2 to 75.9)	76.0 (60.0 to 77.9)	0.53
SvO_2_ (%)					
T0	66.0 (56.8 to 71.4)	68.0 (54.9 to 81.0)	68.1 (57.0 to 71.0)	69.7 (62.1 to 75.9)	0.39
T6	69.0 (63.3 to 72.9)	70.6 (63.4 to 73.8)	71.2 (64.4 to 75.1)	68.0 (58.7 to 74.3)	0.77
Oxygen extraction ratio					
T0	34.7 (28.3 to 44.3)	30.8 (22.7 to 38.4)	29.4 (25.0 to 36.8)	30.7 (24.9 to 37.5)	0.36
T6	29.8 (26.4 to 36.6)	30.2 (24.9 to 31.3)	29.3 (24.1 to 33.3)	30.7 (23.4 to 39.3)	0.68
Pv-aCO_2_ (mmHg)					
T0	8.2 (7.0 to 10.6)*^¶^	1.4 (1.1 to 5.3)*^φ^	6.3 (6.1 to 7.2)^φξ^	2.7 (2.0 to 4.0)^**¶**ξ^	<0.001
T6	7.6 (6.4 to 9.4)**^¶^	8.6 (6.5 to 11.7)^φΨ^	3.5 (0.9 to 4.3)**^φ^	3.3 (2.2 to 4.1)^**¶**Ψ^	<0.001
Pvc-aCO_2_ (mmHg)					
T0	9.7 (7.0 to 12.1)^**¶**^	4.6 (3.3 to 6.1)	7.3 (4.6 to 9.1)	4.4 (2.7 to 5.4)^**¶**^	<0.001
T6	7.0 (5.8 to 9.7)	8.5 (5.6 to 9.6)^Ψ^	4.4 (2.1 to 5.5)	4.0 (1.7 to 5.4)^Ψ^	0.003
**Ventilatory parameters**					
Mechanical ventilation					
T0	24	5	11	23	0.01
T6	24	5	11	23	0.01
PEEP (mmHg), *n*			5.0 (5.0 to 8.0), 11	6.0 (5.0 to 8.0), 23	0.48
T0	6.0 (5.0 to 8.0), 24	5.0 (5.0 to 6.0), 5	5.0 (5.0 to 8.0), 11	8.0 (5.0 to 10.0), 23	
T6	6.5 (5.0 to 8.0), 24	5.0 (5.0 to 7.5), 5			
Tidal volume (ml/kg)					
T0	7.1 (6.3 to 7.7)	7.3 (6.3 to 7.6), 5	7.0 (6.5 to 8.0), 11	7.5 (6.6 to 8.0), 23	0.65
T6	7.2 (6.3 to 7.9)	7.2 (6.6 to 7.6), 5	7.5 (6.8 to 8.0), 11	7.5 (6.9 to 8.0), 23	0.65

Data presented as mean (25 to 75% interquartile range). *CVP*, central venous pressure; FiO_2_, oxygen inspired fraction; *MAP*, mean arterial pressure; PaCO_2_, arterial carbon dioxyde pressure; PaO2, arterial oxygen pressure; *PAOP*, pulmonary artery occlusion pressure; PEEP, positive end-expiratory pressure; *Pv-aCO*_*2*_, mixed venous-to-arterial carbon dioxide difference; *Pvc-aCO*_*2*_, central venous-to-arterial carbon dioxide difference; *PvcCO*_*2*_, central venous carbon dioxide pressure; *PvmCO*_*2*_, mixed venous carbon dioxide pressure; *ScvO*_*2*_, central venous oxygen saturation; *SvO*_*2*_, mixed venous oxygen saturation; T0, time 0; T6, 6 hours after Time 0. *P <0.05 for Groups 1 vs. 2; **P <0.05 for Groups 1 vs. 3; ^¶^*P* <0.05 for Groups 1 vs. 4; ^φ^*P* <0.05 for Groups 2 vs. 3; ^Ψ^*P* <0.05 for Groups 2 vs. 4; and ^ξ^*P* <0.05 for Groups 3 vs. 4.

**Figure 4:**
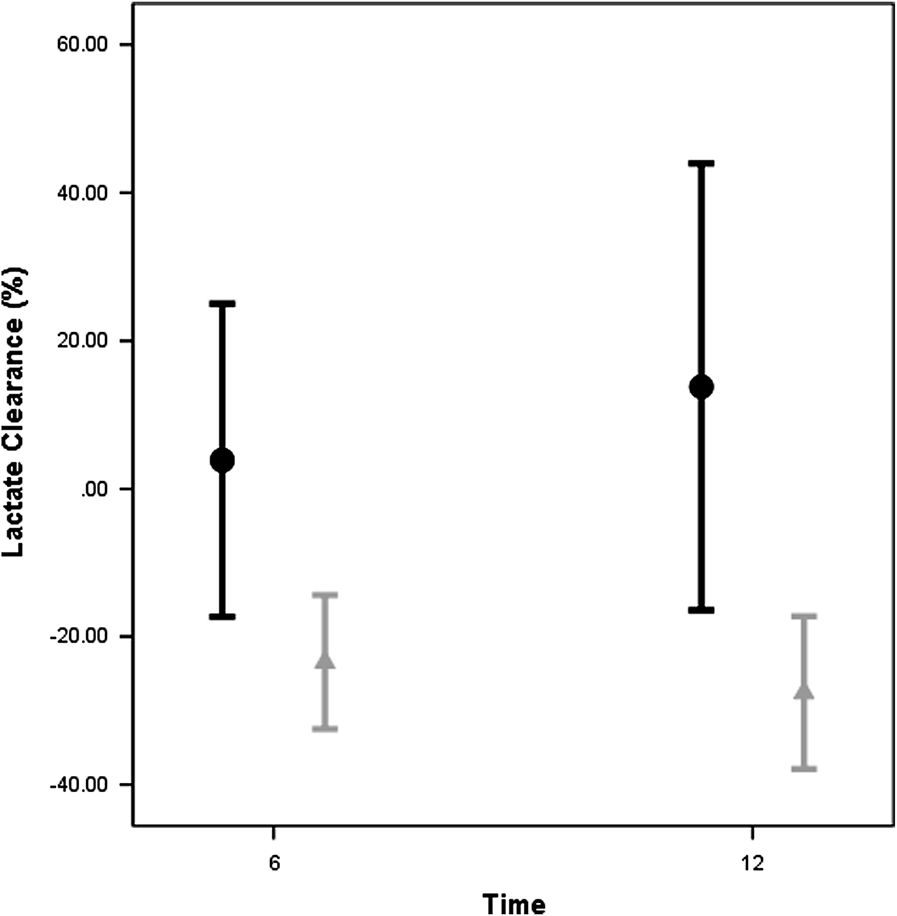
**Lactate clearance (%) 6 and 12 hours after Time 0 for patients with normal or high mixed venous-to-arterial carbon dioxide difference at 6 hours.** Significant differences for lactate clearance (Time 0 (T0) to 6 hours later (T6) and T0 to 12 hours later (T12)) between patients with persistently high (that is, high-to-high and normal-to-high groups) and normalized mixed venous-to-arterial carbon dioxide difference at T6 (high-to-normal and persistently low groups). A negative percent clearance indicates a reduction in lactate levels. Black bars: High Pv-aCO_2_ at T6; Gray bars: Low Pv-aCO_2_ at T6.

**Figure 5:**
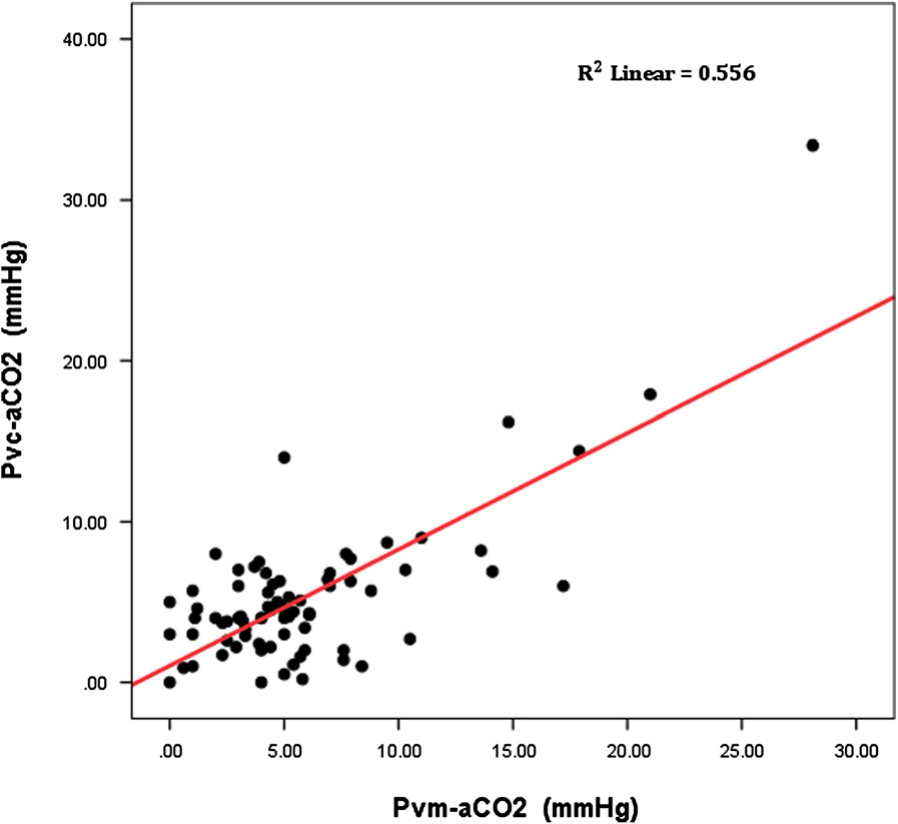
**Correlation between mixed venous carbon dioxide pressure and central venous-to-arterial carbon dioxide difference.** Scatter plot representing the mixed-venous to arterial carbon dioxide (Pvm-aCO_2_) difference versus the central-venous to arterial carbon dioxide difference (Pvc-aCO_2_). Pearson correlation: 0.71 (95% confidence interval: 0.47 to 0.86); *R*^2^ = 0.55, *P* <0.001.

Patients with elevated Pv-aCO_2_ at T6 had slower lactate clearances at T6 and T12 than patients attaining a normal Pv-aCO_2_ during the first 6 hours of resuscitation (Figure 4). We also observed a significant linear correlation between mixed-venous to arterial pCO_2_ and central-venous to arterial pCO_2_ (Pearson correlation: 0.71, 95% confidence interval: 0.47 to 0.86; *P* <0.001) but with moderate agreement between them (*R*^2^ = 0.556, *P* <0.001) (Figure 5). Additionally, significant differences were observed for the time course of Pv-aCO_2_ and central venous-to-arterial carbon dioxide difference (Pvc-aCO_2_) during the first 24 hours for survivor and nonsurvivors at day 28 (repeated-measures analysis of variance, *P* = 0.003 and *P* = 0.03, respectively; Figure S4a,b in Additional file 1).

Finally, patients who achieved ScvO2?≥?70% or SvO2?≥?65% but maintained high Pv-aCO2 at T0, T6 and T12 had a higher mortality risk at Day 28 (Table 3).

**Table 3:** Mortality risk ratio for patients with mixed oxygen saturation ≥65% but with mixed venous-to-arterial carbon dioxide difference ≥6 mmHg at day 28

**Time**	**Relative risk**^**a**^	**Confidence interval**	***P *** **value**
0 hours	1.77	0.97–3.22	0.06
6 hours	2.23	1.20–4.13	0.01
12 hours	2.41	1.42–4.10	0.001

^*a*^*R*elative mortality risk at day 28.

## Discussion

We studied a cohort of patients during the very early phases of septic shock who were subjected to a comprehensive resuscitation aimed to target the usual hemodynamic and oxygen metabolism parameters. A recent study demonstrated how Pv-aCO_2_ could be a tool to detect persistent inadequate resuscitation during septic shock [18] although it was not conducted during very early stages of resuscitation. Even a faster enrollment, our study showed that a number of patients had approximately normal SvO_2_ and ScvO_2_ at catheter insertion as it has been reported at ICU admission [22], and most of them reached normal oxygen-derived parameters at 6 hours. However, despite attaining the SvO_2_ and ScvO_2_ targets (and after adjusting for SvO_2_) and an apparent global hemodynamic normalization in most patients, those with persistently high Pv-aCO_2_ developed more severe multiorgan dysfunction at day 3 than patients evolving with normal Pv-aCO_2_ during the first 6 hours of resuscitation or those who evolved from high to normal PvaCO_2_. Additionally, we observed that persistently high Pv-aCO_2_ was associated with a lower survival at day 28.

Venous hypercarbia is a marker of limited blood flow during cardiac arrest and shock states [8-19]. Recent observations have suggested that Pv-aCO_2_ might identify septic patients who remain inadequately resuscitated despite achieving ScvO_2_ goals [15]. Consistent with these findings, we found that patients in septic shock achieving ScvO_2_ ≥70% or SvO_2_ ≥65% had worse outcomes when a concomitant high Pv-aCO_2_ was observed. These data reinforce the idea that Pv-aCO_2_ provides additional information to hemodynamic and oxygen parameters habitually used during resuscitation of septic shock. Nevertheless, the underlying mechanisms that explain increases in Pv-aCO_2_ during septic shock are incompletely understood; however, to current knowledge, an increased Pv-aCO_2_ results from the interactions between blood flow to the tissues, aerobic and anaerobic CO_2_ generation, and the CO_2_ dissociation curve.

According to the Fick equation, during steady state the CO_2_ excretion equals the product of cardiac output by the difference between mixed venous blood CO_2_ content and arterial blood CO_2_ content. Some studies have emphasized on the key role of cardiac output on venous to arterial CO_2_ content differences and indeed a curvilinear relationship between these two variables has been described [14]. However, in our study we found a poorer concordance between cardiac output and Pv-aCO_2_ at each time point of resuscitation (Figure 3) and, in fact, the cardiac output remained normal or even high during the first 24 hours of resuscitation (Figure S2 in Additional file 1), suggesting some independence between Pv-aCO_2_ and macrovascular blood flow changes. While in non-inflammatory low-flow states tissue and regional hypercarbia can be easily explained by the CO_2_ stagnation phenomenon [15,16], the interpretation of an increased tissue and/or regional CO_2_ during inflammatory conditions is more complex. Sepsis may thereby be associated with the coexistence of normal or even high cardiac output, inter-organ and intra-organ blood flow redistribution, and altered microvascular and oxygen extraction capabilities. All of these alterations can influence the tissue CO_2_ production and elimination.

A study by Neviere and colleagues thus demonstrated the key role of microvascular blood flow on gastric CO_2_ accumulation [27]. Similarly, using simultaneous gastric tonometry and laser Doppler flowmetry, Elizalde and colleagues demonstrated the association between gastric mucosal pH and mucosal blood flow, regardless of macrohemodynamic variations [28]. Likewise, Tugtekin and colleagues demonstrated in a porcine sepsis model that the increase of mucosal to arterial CO_2_ gap was related to the heterogeneity of gut mucosal blood flow, even though cardiac output and mesenteric blood flow were maintained [29]. Meanwhile, Creteur and colleagues found a significant correlation among sublingual CO_2_, gastric mucosal CO_2_ and microcirculatory heterogeneity in human septic shock during dobutamine infusion, and suggested that the primary determinant of tissue CO_2_ accumulation was the microcirculatory blood flow [30]. Hence, there is an evident link between blood flow and tissue or local CO_2_ accumulations conducting to increase tissue or venous-to-arterial CO_2_ differences, but sometimes normal macrohemodynamics does not prevent elevation of Pv-aCO_2_. The near normalization of the oxygen and hemodynamic parameters between the subgroups in our study suggests that venous CO_2_ accumulation encloses more complex mechanisms than macrovascular stagnation, and we could hypothesize that microvascular blood flow distribution is one of several factors potentially influencing the behavior of Pv-aCO_2_ during inflammatory conditions in which the heterogeneity of microvascular blood flow is increased. However, this hypothesis should be confirmed in future studies.

The interpretation of hyperlactatemia in sepsis is very complex, especially in septic shock [31] since anaerobic metabolism, non-anaerobic generation and slow clearance can conduct lactate accumulation. We observed higher lactate levels and slower lactate clearance at T6 and T12 in patients with persistently high Pv-aCO_2_ during the first 6 hours of resuscitation. Interestingly, Pv-aCO_2_ (and Pvc-aCO_2_) kinetics seems to anticipate a slower lactate clearance (Figure S5 in Additional file 1). A high Pv-aCO_2_ could indicate a decrease in global or microvascular blood flow conducting to slow lactate clearance. However, a high Pv-aCO_2_ could also reflect the persistence of anaerobic metabolism as result of bicarbonate buffering of protons derived from fixed acids [32]. Thus, an increased Pv-aCO_2_ to oxygen consumption ratio could reflect global anaerobic metabolism as was proposed by Mekontso-Dessap and colleagues [19]. However, even in the presence of anaerobic metabolism, a high efferent venous blood flow could be sufficient to wash out the global CO_2_ generation from the hypoperfused peripheral tissues and, in this case, Pv-aCO_2_ could not increase. In fact, hypoperfusion could persist in some of our patients and even oxygen parameters, global hemodynamics or Pv-aCO_2_ remain normal.

Finally, we found a significant linear correlation but moderate agreement between venous–arterial CO_2_ differences obtained from mixed venous and central venous samples that agree with recent observations published simultaneously to the review of our paper [21]. Even though Pvc-aCO_2_ can be easily obtained and speedily usable in the emergency room, the point about whether Pvc-aCO_2_ and mixed venous carbon dioxide pressure (Pv-aCO_2_ in our study) are really interchangeable should be addressed in future studies.

Our study has some limitations. First, our observations are restricted to macro-hemodynamic variables, and Pv-aCO_2_ is another global variable that does not necessarily represent tissue or regional vascular perfusion at different beds. We did not describe regional perfusion variables as gastric tonometry or local tissue CO_2_ accumulation; hence, normal Pv-aCO_2_ might also occur when regional hypoperfusion is ongoing.

Second, we suggest that persistently high Pv-aCO_2_ reflects tissue or regional hypoperfusion. We hypothesized that Pv-aCO_2_ could reflect the venous CO_2_ accumulation due to the heterogeneous microcirculatory blood flow when cardiac output and oxygen parameters remain normal or even high or, eventually, Pv-aCO_2_ could reflect anaerobic CO_2_ generation. However, mechanism conducting to venous CO_2_ accumulation during inflammatory conditions should be explored in future studies.

Third, during conditions of tissue hypoxia but with preserved blood flow (even though during anaerobic metabolism carbon dioxide production - VCO_2_ - decreases less than oxygen consumption -VO_2_-), venous blood flow might be high enough to ensure adequate washout of the CO_2_ produced by hypoxic cells, thereby preventing a Pv-aCO_2_ increase.

Fourth, we assumed that a linear relationship exist between partial CO_2_ pressure and CO_2_ content at the venous and arterial levels [33,34]. Pv-aCO_2_ could thus be used as a surrogate for the Cv-aCO_2_. However, previous research has shown that the Haldane effect causes paradoxical increases in Pv-aCO_2_ during blood flow increases [33,34]. Unfortunately, the calculation of CO_2_ content is complex and subject to errors due to the number of variables included in the formulas. Simplified formulas are easy to use, but wide differences in venous and arterial acid–base status (for example, ischemic hypoxia) can preclude their use. Nevertheless, some authors consider that the Haldane effect exerts a minor influence, and in most cases Pv-aCO_2_ and CO_2_ content differences develop similarly [35].

Finally, our observations were restricted to a small sample of patients in septic shock. Although our findings seem logical and biologically plausible, they should be confirmed in future studies.

## Conclusions

The persistence of high Pv-aCO_2_ during the early resuscitation of patients in septic shock is associated with significant higher multiorgan dysfunction and poor outcomes. Although underlying mechanisms that increase Pv-aCO_2_ among patients in septic shock must be clarified, Pv-aCO_2_ might identify a high risk of death in apparently resuscitated patients. Future studies should test Pv-aCO_2_ as a perfusion goal during early phases of the resuscitation of patients in septic shock.

## Key messages

• Persistent high Pv-aCO_2_ is related to more severe multiorgan dysfunction and worse outcomes in apparently resuscitated septic shock patients.

• Mechanisms conducting to increase Pv-aCO_2_ during inflammatory conditions are insufficiently understood. Variations in Pv-aCO_2_ were independent of macro-flow variables (that is, cardiac output) or oxygen metabolism targets (that is, SvO2, oxygen extraction rate), suggesting that venous stagnation is not the single explanation for venous CO_2_ accumulation.

• Pv-aCO_2_ might identify a high risk of death in apparently resuscitated septic shock patients, and could be explored as a tissue perfusion goal during resuscitation as Pv-aCO_2_ tracks ischemic hypoxia.

## Abbreviations

CO2: Carbon dioxide; Pv-aCO2: Mixed venous-to-arterial carbon dioxide difference; Pvc-aCO2: Central venous-to-arterial carbon dioxide difference; ScvO2: Central venous oxygen saturation; SvO2: Mixed venous oxygen saturation.

## Competing interests

The authors declare they have no competing interests.

## Authors’ contributions

GAO-T contributed to the study conception, design and manuscript preparation. DFB-R, MU, JDT, AG and WB were involved in data collection and revising the manuscript. AFG, MG, CA-D and GH revised the manuscript critically. All authors read and approved the final manuscript.

## Supplementary Material

Additional file 1 **Figure S1a presenting the time course of ScvO2 (%) during the first 24 hours for survivors and nonsurvivors at day-28. Figure S1b** presenting the time course of SvO2 (%) during the first 24 hours for survivors and nonsurvivors at day 28. **Figure S2** presenting the time course of cardiac output (l/minute) during the first 24 hours for survivors and nonsurvivors at day 28. **Figure S3** presenting the scatter plot between cardiac index and Pv-aCO_2_ (according to Pv-aCO_2_ at T6). **Figure S4a** presenting the time course of Pv-aCO2 (mmHg) during the first 24 hours for survivors and nonsurvivors at day 28. **Figure S4b** presenting the time course of Pvc-aCO2 (mmHg) during the first 24 hours for survivors and nonsurvivors at day 28. **Figure S5** presenting the time course of lactate levels (mmol/l) during the first 24 hours for survivors and nonsurvivors at day 28. Click here for file
