# Supplementary material for: Persistently high venous-to-arterial carbon dioxide differences during early resuscitation are associated with poor outcomes in septic shock
Source: Crit Care. 2013 Dec 13;17(6):R294. doi: 10.1186/cc13160 (PMC4056748; doi:10.1186/cc13160)

**Persistently high venous-to-arterial carbon dioxide differences during early resuscitation are associated with poor outcomes in septic shock**

**Gustavo A. Ospina-Tascón ^1, 2^, Diego F. Bautista-Rincón ^1^, Mauricio Umaña ^1^, José D. Tafur ^1^, Alejandro Gutiérrez ^1^, Alberto F. García ^1^, William Bermúdez ^2^, Marcela Granados ^1^, César Arango-Dávila ^2^, and Glenn Hernández ^3^**

1. Intensive Care Unit, Fundación Valle del Lili, Cali, Colombia
2. Universidad ICESI, Biomédica Research Group,Cali, Colombia
3. Departamento de Medicina Intensiva, Pontiﬁcia Universidad Catolica de Chile. Santiago de Chile, Chile.

**SUPPLEMENTARY MATERIAL**

**Results**

Some additional results are presented below.

**Figure S1a. Time-course of ScvO2 (%) during the first 24 hours for survivors and non-survivors at day-28.**

Repeated measures analysis of variance, p=0.22.

**Figure S1b. Time-course of SvO2 (%) during the first 24 hours for survivors and non-survivors at day-28.**

Repeated measures analysis of variance, p=0.65.

**Figure S2. Time-course of cardiac output (L/min) during the first 24 hours for survivors and non-survivors at day-28.**

Repeated measures analysis of variance, p=0.37.

**Figure S3. Scatter plot between cardiac index and Pv-aCO_2_ (according to Pv-aCO_2_ at T6)**

Blue points: Pv-aCO_2_ < 6 mmHg; Red points: Pv-aCO_2_ ≥ 6 mmHg

**Figure S4a. Time-course of Pv-aCO2 (mmHg) during the first 24 hours for survivors and non-survivors at day-28.**

Repeated measures analysis of variance, p=0.003. Pv-aCO_2_ denotes mixed-venous to arterial pCO_2_ difference.

**Figure S4b. Time-course of Pvc-aCO2 (mmHg) during the first 24 hours for survivors and non-survivors at day-28.**

Repeated measures analysis of variance, p=0.03. Pvc-aCO_2_ denotes central-venous to arterial pCO_2_ difference.

**Figure S5. Time-course of lactate levels (mmol/L) during the first 24 hours for survivors and non-survivors at day-28.**

Repeated measures analysis of variance, p<0.0001.

**Figure S1a.**


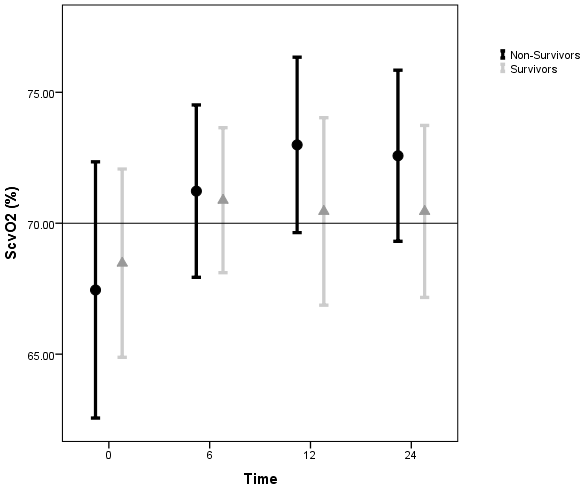


**Figure S1b.**


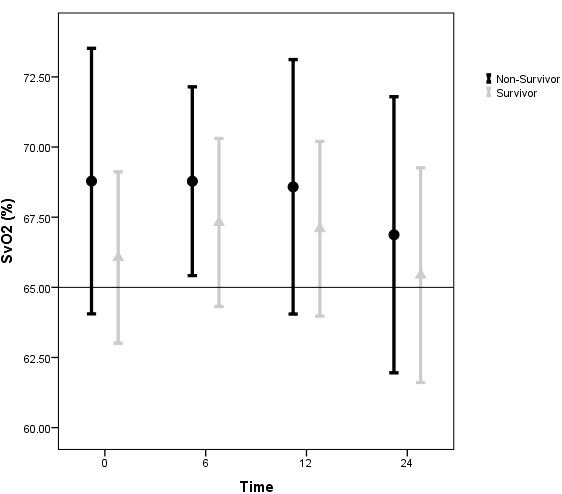


**Figure S2.**


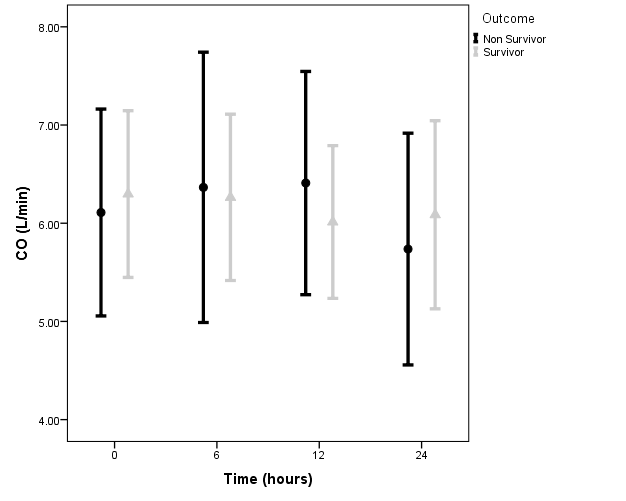


**Figure S3.**

**
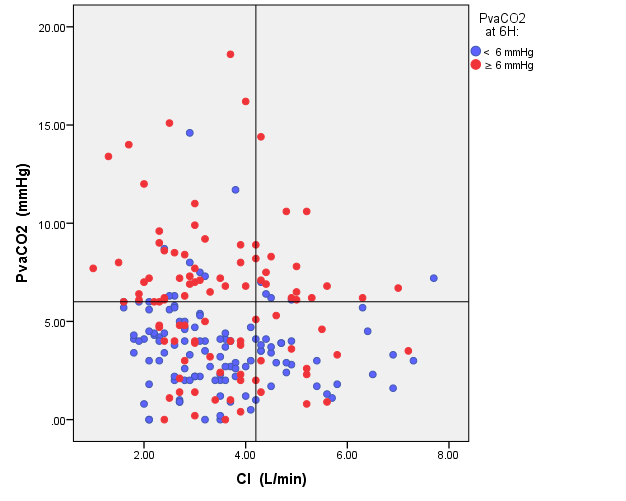
**

**Figure S4a.**


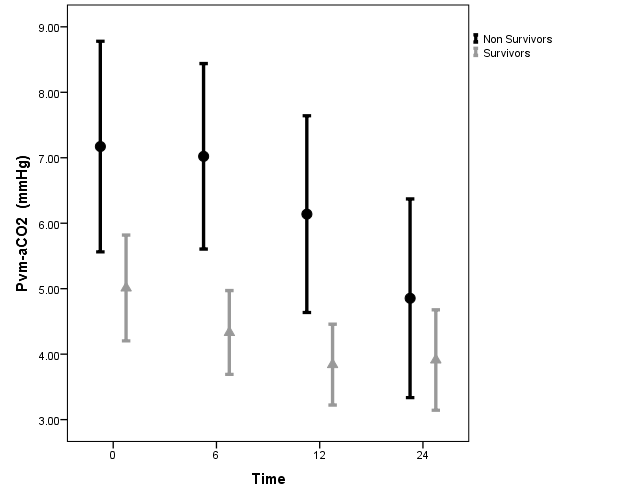


**Figure S4b.**


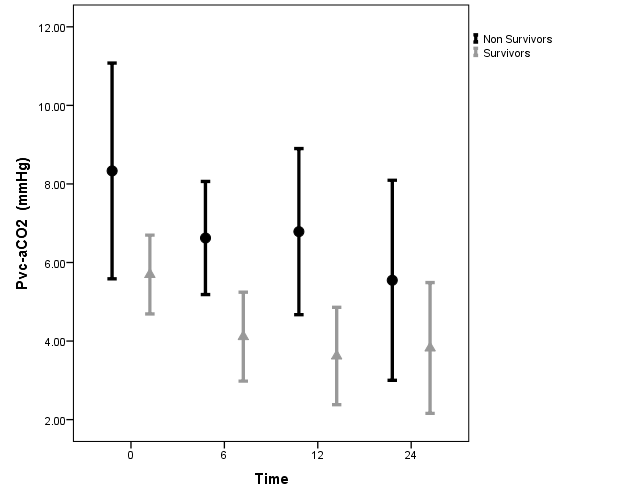


**Figure S5.**


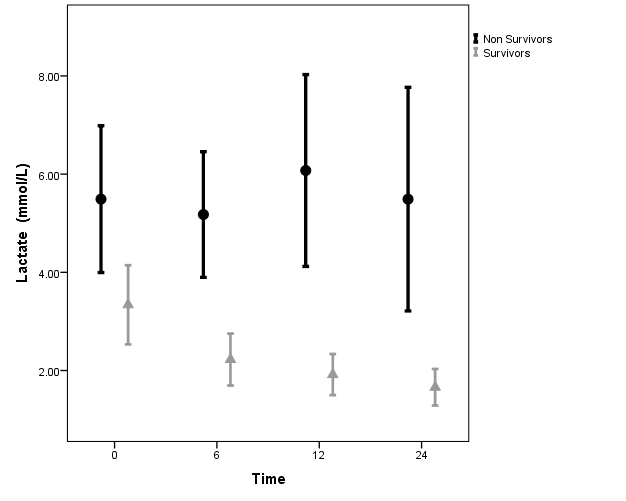

Supplement: Additional file 1 — Figure S1a presenting the time course of ScvO2 (%) during the first 24 hours for survivors and nonsurvivors at day-28. Figure S1b presenting the time course of SvO2 (%) during the first 24 hours for survivors and nonsurvivors at day 28. Figure S2 presenting the time course of cardiac output (l/minute) during the first 24 hours for survivors and nonsurvivors at day 28. Figure S3 presenting the scatter plot between cardiac index and Pv-aCO2 (according to Pv-aCO2 at T6). Figure S4a presenting the time course of Pv-aCO2 (mmHg) during the first 24 hours for survivors and nonsurvivors at day 28. Figure S4b presenting the time course of Pvc-aCO2 (mmHg) during the first 24 hours for survivors and nonsurvivors at day 28. Figure S5 presenting the time course of lactate levels (mmol/l) during the first 24 hours for survivors and nonsurvivors at day 28. [file cc13160-S1.docx]
